# NMR-based metabolic profiling of children with premature adrenarche

**DOI:** 10.1007/s11306-022-01941-4

**Published:** 2022-10-14

**Authors:** Konstantina Matzarapi, Aristeidis Giannakopoulos, Styliani A. Chasapi, Dimitra Kritikou, Alexandra Efthymiadou, Dionisios Chrysis, Georgios A. Spyroulias

**Affiliations:** 1 Department of Pharmacy, School of Health Sciences, University of Patras, 26504 Rio, Greece; 2 Division of Endocrinology Department of Pediatrics, Medical School, University of Patras, 26504 Rio, Greece

**Keywords:** Premature adrenarche, Metabolomics, NMR spectroscopy, Metabolomic profiling, Biofluids

## Abstract

**Introduction:**

Premature adrenarche (PA) for long time was considered a benign condition but later has been connected to various diseases in childhood and adulthood which remains controversial.

**Objective:**

To investigate the effect of premature adrenarche on the metabolic phenotype, and correlate the clinical and biochemical data with the metabolic profile of children with PA.

**Methods:**

Nuclear magnetic resonance (NMR)-based untargeted and targeted metabolomic approach in combination with multivariate and univariate statistical analysis applied to study the metabolic profiles of children with PA. Plasma, serum, and urine samples were collected from fifty-two children with Idiopathic PA and forty-eight age-matched controls from the division of Pediatric Endocrinology of the University Hospital of Patras were enrolled.

**Results:**

Metabolomic results showed that plasma and serum glucose, myo-inositol, amino acids, a population of unsaturated lipids, and esterified cholesterol were higher and significantly different in PA children. In the metabolic profiles of children with PA and age-matched control group a gradual increase of glucose and myo-inositol levels was observed in serum and plasma, which was positively correlated their body mass index standard deviation score (BMI SDS) values respectively. Urine ^1^H NMR metabolic fingerprint of PA children showed positive correlation and a clustering-dependent relationship with their BMI and bone age (BA) respectively.

**Conclusion:**

This study provides evidence that PA driven metabolic changes begin during the childhood and PA may has an inductive role in a BMI–driven increase of specific metabolites. Finally, urine may be considered as the best biofluid for identification of the PA metabolism as it reflects more clearly the PA metabolic fingerprint.

**Supplementary Information:**

The online version contains supplementary material available at 10.1007/s11306-022-01941-4.

## Introduction

Idiopathic Premature adrenarche (PA) has been considered, for a long time, to be a benign condition when pathological causes of hyperandrogenism such as adrenal tumors and non-classical adrenal hyperplasia are excluded. Risk factors linked to PA, are prematurity, birth weight, rapid weight gain and obesity (Novello & Speiser, 2018; Voutilainen & Jääskeläinen, 2015).

Furthermore, in recent years, many studies have proposed the association of PA with insulin resistance, increased cardiovascular risk, metabolic syndrome, ovarian hyperandrogenism and increased risk for polycystic ovarian syndrome (PCOS), especially in girls with PA and low birth weight (Ibáñez et al., 1998; Neville & Walker, 2005). However, the impact of PA as a precursor of metabolic syndrome of childhood remains controversial, since there are published studies that do not support the link of PA with low birth weight (Boonstra et al., 2004) or the later sequela of ovarian hyperandrogenism (Mathew et al., 2002). Until now, the biochemical definition of premature adrenarche is based on a modest increase of adrenal androgens in blood serum. To give a deeper insight into the underlying pathophysiological processes of PA, we studied the metabolic profiles of this condition, using spectroscopic tools on diverse biological fluids (Georgiopoulou et al., 2022; Holmes et al., 2008).

More specifically, we performed nuclear magnetic resonance (NMR)-based untargeted metabolomic analysis on three biofluids (plasma, serum, and urine) of children with PA and a control group, focusing mainly on low molecular weight metabolites (< 1 kDa) (Chasapi et al., 2019; Georgakopoulou et al., 2020; Katsila et al., 2021; Tsagkarakou et al., 2021; Zompra et al., 2021). Considering that children with PA have their own steroid metabolic pattern (Remer et al., 2005; Storbeck et al., 2019), we examined a standard group of steroid hormones which are commonly used in clinical practice as markers of their PA clinical characteristics. The three measured adrenal steroids dehydroepiandrosterone sulfate (DHEAS), androstenedione (Δ4Α) and 17α-hydroxyprogesterone (17-OHP) were examined as categorizing factors of the ^1^H NMR metabolic profiles of children with PA. Moreover, we evaluated the association of bone age (BA), body mass index (BMI) standard deviation score (SDS) and birth weight (BW) to the NMR metabolic profiles.

## Methods

### Ethics statement

The study was approved by the Ethics Committee of the University Regional General Hospital of Patras, Rio, Greece. Informed consent obtained from all the participants of the study. All methods performed in accordance with the Ethical Principles for Medical Research Involving Human Subjects of the Helsinki Declaration.

### Patient’s population

A total of 100 children, 52 with PA (49 girls and 3 boys) and 48 healthy children (30 girls and 18 boys) were enrolled in this study (Table 1). All children with PA had onset of signs of adrenarche before the age of 8 years for girls and 9 years for boys, with no other adrenal pathology as the cause of adrenarche. Girls with thelarche or clitoromegaly and boys with testes > 4 mL were excluded from the study. All patients were followed for at least 6 months, and none entered puberty. None of the PA children and control subjects was on any medication. Birth weight and length were recorded from the national book of child’s health. The control group consisted of girls and boys attending our community pediatric outpatient clinic for routine health visits, or for preoperative assessment for minor surgeries. Age, height (measured by a Harpenden stadiometer) and weight were measured, and pubertal status was clinically assessed according to Tanner stages (Emmanuel & Bokor, 2021). ΒΑ was assessed by the same senior pediatric endocrinologist (D. Chrysis) and evaluated according to Greulich and Pyle atlas (Dahlberg et al., 2019). The study was approved by the Ethics Committee of the University Hospital of Patras. Informed consent was obtained from the parents of the participants.

**Table 1:** Baseline characteristics of 100 participants

Characteristics	Controls n = 48	PA n = 52	P-value
Male/Female	18/30	3/49	P < 0.001^*^
Age (years ± SD)	7.69 ± 2.17	7.72 ± 1.56	P = 0.93^**^
Gestational age SGA/AGA/LGA	7/38/1	6/46/0	P = 0.55^**^
Birth weight (gr ± SD)	2891.05 ± 739.99	2965.5 ± 440.42	P = 0.54^**^
BMI: > 1.5SD/ ≤ 1.5SD	n:7/n:41	n:19/n:33	
DHEAS (mcg/dL)	–	114.69 ± 52.85	
Δ4A (ng/mL)	–	0.46 ± 0.31	
17-OH Progesterone (mcg/mL)	–	1.18 ± 0.75	
Bone age (year)	–	Median = + 1 y, range = 0 y to 3 y	

*SGA* small for gestational age, *AGA* appropriate for gestational age, *LGA* large for gestational age, *BW* birth weight, *BMI* body mass index, *DHEAS* dehydroepiandrosterone sulfate, *17-OHP* 17a-hydroxyprogesterone, *Δ4A* androstenedione, *BAA* bone age advancement

^*^Testing for gender performed using Chi-squared test, **Testing for age, gestational age and birth weight performed using t-test

### Sample collection and preparation

Biofluids (plasma, serum, and urine) were collected, stored, and prepared for NMR analysis according to the below-mentioned protocols, with adaptations as described by Bernini et al. (Bernini et al., 2011). Storage and NMR sample preparation were carried out at the Department of Pharmacy of University of Patras.

Fasting blood plasma and serum samples were withdrawn from donors using BD Vacutainer® K3-EDTA (K3-ethylenediamine tetra-acetate) spray-coated tubes and BD Vacutainer® SST™, respectively. Serum samples were left 0.5 h at room temperature (RT) for the completion of the clotting cascade. All blood samples were centrifuged at 1500 g × 0.17 h at RT and supernatant from each sample was aliquoted in fractions of 600 μL. Aliquots were stored in 2 mL cryovials at -80 °C. Urine samples were collected in fasting state and were stored immediately at 4 °C for a maximum of 2 h before their centrifugation at 2500 g × 0.17 h at RT. Supernatant from each sample was aliquoted in three fractions of 1 mL and they were stored in 2 mL cryovials at − 80 °C.

### NMR sample preparation

NMR samples were prepared by thawing aliquots of the stored fluids followed by the addition of a suitable buffer. Plasma and serum samples were mixed with a buffer of pH = 7.4 (0.14 M Na_2_HPO_4_, 0.5 mM 4,4-dimethyl-4-silapentane-1-sulfonic acid (DSS), 4% NaN_3_ in H_2_O) according to protocols reported elsewhere Buffer used for urine samples prepared in 100% D_2_O and pH = 7.4 (1.5 M KH_2_PO_4_, 0.05 mM DSS and 4% NaN_3_ 2 mM). 300 μL of plasma and serum samples were mixed with 240 μL buffer and 60 μL D_2_O, while 540 μL of urine samples were mixed with 60 μL buffer (Suarez-Diez et al., 2017). The final step before NMR experiments was the transfer of 550 μL in a 5 mm NMR tube (Bruker BioSpin GmbH).

### NMR experiments

All samples were measured in a Bruker Avance III HD 700 MHz NMR spectrometer equipped with a 5 mm cryogenically cooled TCI gradient probe, at 36.85 °C and 26.85 °C for blood derivatives and urine, respectively and were processed using TopSpin 3.5 pl7 software (Bruker BioSpin GmbH). One-dimensional ^1^H NOESY (Nuclear Overhauser Effect SpectroscopY) spectra using presaturation routine for water suppression were recorded, with 32 number of scans of 98.3 K data points with 14,005.6 Hz spectral width, for plasma and serum. For urine samples 1D ^1^H NOESY spectra recorded with 64 scans of 65.5 K data points and 10,504.20 Hz spectral width. 1D ^1^H CPMG (Carr-Purcell-Meiboom-Gill) spectra were acquired only for plasma and serum samples to avoid the signal contribution, result of the high molecular weight molecules (protein and lipid content) in their composition. For the acquisition of the 1D ^1^H CPMG spectra, 32 scans of 73.7 K data points and 18,028.8 Hz spectral width were used. Homonuclear 2D NMR ^1^H *J*-resolved (*J*-res) spectra were also acquired for all each sample using four scans per 128 increments for F1 (spin–spin coupling constant axis) and 12.3 K data points for F2 (chemical shift axis). Spectral width used was 78.13 Hz and 11,627.91 Hz for F1 and F2, respectively. During all experiments, the signal of water was suppressed by presaturation pulse.

### NMR spectral processing

Before the statistical analysis, zero- and first-order phase correction of spectra was applied. The plasma and serum spectra were aligned on the double resonance peak of anomeric proton of a-glucose at 5.24 ppm, while urine spectra were aligned on the DSS singlet at 0.00 ppm (Pearce et al., 2008) using TopSpin 4.0.7 (Bruker BioSpin GmbH). Both procedures were performed manually. 8 spectra out of the 300, were not suitable for the analysis and were excluded (Table 1). The NMR spectral data were converted by AMIX software (Bruker BioSpin GmbH) in bucket tables. Spectral regions 0.00–10.00 ppm segmented into integrated spectral domains (buckets) of equal width (0.02 ppm) excluding the water signal. Furthermore, ^1^H signals of the anticoagulant Ethylenediamine tetra-acetate (EDTA) (H-EDTA, Ca^2+^-EDTA, Mg^2+^-EDTA) were excluded from the bucketing of plasma data, while DSS and Urea resonance signals were excluded from the bucketing of urine data.

### Statistical analysis and computational tools

The metabolites’ assignment was performed in a non-automated way using the free version of Chenomx NMR Suite 8.3, the databases Human Metabolome DataBase (HMDB), Biological Magnetic Resonance Bank (BMRB), DrugBank and the available bibliography (Salek et al., 2007; Wishart et al., 2007). A few ^1^H signals could not be assigned due to the high percentage of overlapping areas. SIMCA 16.0.1 (Umetrics, Sweden) and the programming language R (Rstudio 3.5.2) were used for the multivariate (untargeted) and univariate (targeted) statistical analysis of the spectral data (Saccenti et al., 2014). Pareto scaling was selected as the most suitable scaling method for the blood samples, and urine samples were scaled to unit variance. The unsupervised multivariate method principal component analysis (PCA) was applied to all NMR metabolomic profiles, as a data structure investigation approach (data not shown). It was followed by the supervised multivariate method partial least square—discriminant analysis (PLS-DA) to provide information about each sample’s class. Each PLS-DA model was constructed using the first six latent variables (LVs). PLS-DA VIP scores above 1.00 reveal the statistically significant metabolites for the examined classification. Additionally, supervised partial least square (PLS) models were constructed to relate metabolic profiles for continuous response to androgens, BA, BMI (expressed as SD scores-SDS) and BW. Androgen levels and BA associations to PA metabolic profile were estimated through the population distribution in each PLS model. The parameters R^2^ and Q^2^ were calculated via sevenfold cross validation, summarizing the appropriateness of the model for the examined data sets. Metabolite univariate analysis and statistical significance were based on the non-parametric statistical test Kruskal–Wallis by ranks (H test) for independent samples at level of significance a = 0.05. For each one successfully assigned metabolite, all spectra were aligned in the peak of interest and the area under the curve was calculated (Krzywinski & Altman, 2014). The false discovery rate (FDR) correction was applied to the Kruskal–Wallis test results according to the Benjamini & Hochberg method and the mean log_2_-fold changes were also calculated for the examined metabolites (Benjamini & Hochberg, 2000; Ren et al., 2015). The investigation of the metabolite-metabolite correlations between the two biofluids (urine and plasma) conducted using the Correlation Heatmaps tool for features on MetaboAnalyst 5.0 platform (Pang et al., 2021).

### Data deposition

NMR data have been uploaded in the MetaboLights database (https://www.ebi.ac.uk/metabolights) (Haug et al., 2013). Study’s accession number is MTBLS2387.

## Results

In total, 39 metabolites were identified in plasma, 38 metabolites in serum and 54 metabolites in urine ^1^H NMR spectra (Matzarapi et al., 2021). Blood plasma and serum metabolic profiles did not diverge much on the metabolites’ composition but differed from urine metabolome, as expected. Urine metabolome due to the inherent high chemical complexity contributes significantly to the metabolic signature of PA.

The comprehensive multivariate and univariate statistical analysis of the ^1^H NMR metabolic profiles of children with PA and age-matched controls were performed for the three examined biofluids. PLS-DA classification models revealed a clustering tendency of plasma, serum, and urine ^1^H NMR metabolic profiles of children diagnosed with PA compared to the controls. Univariate statistical analysis of targeted metabolite levels reveals smooth differences between the PA and the control’s metabolic profile. Investigation of the association level amongst the metabolite bulk identified in the two biofluids, urine and plasma, performed through interactive analysis and using linear regression method. Pearson’s correlations of the 39 plasma and the 54 urine metabolites on the semiquantitative and log-transformed data were measured and illustrated (Fig S1). Correlation pattern revealed two major clusters, which are considered to exhibit high consistency by analogy to the biofluid of origin. According to this outcome and due to accurate biological inferences, all NMR metabolomic data were analysed and estimated independently.

### Plasma and serum samples

**Fig. 1:**
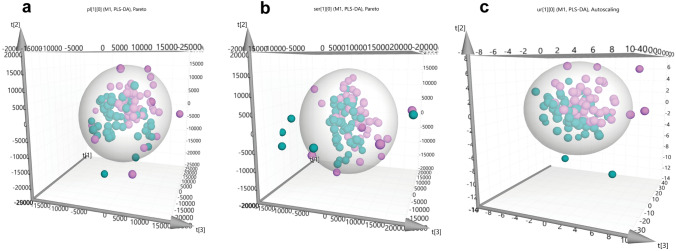
NMR-based non targeted analysis of plasma, serum, and urine PA metabolic profile. PLS-DA score plots of the three first components of the statistically significant comparisons among children. **a** ^1^H CPMG NMR plasma spectra of 50 PA versus the 48 controls; **b** ^1^H CPMG NMR serum of 51 PA versus the 47 controls; **c** ^1^H 1D NOESY NMR urine of 51 PA and 45 control. Children with PA are presented with turquoise, while control children are with purple spheres

Plasma metabolic profiles reveal high similarity in the PLS-DA multivariate space (R^2^Y (cum) = 33.5% and Q^2^Y (cum) = − 47%) (Fig. 1a). Between children with PA and controls, levels of glucose, glycerol, serine, alanine, lysine, unsaturated fatty acids and esterified cholesterol were found to be statistically significant, according to the PLS-DA variable importance in projection (VIP) scores, for the classification of both plasma and serum metabolic profiles (Table 2). The metabolites leucine, lactic acid and *N*-acetyl-D-glucosamine (GlcNAc) were also statistically significant for the diagnostic classification of plasma samples (Table 2). Also, serum metabolic profiles when used for classification displayed minimum clustering tendency (Fig. 1a R^2^Y (cum) = 45.6% and Q^2^Y (cum) = − 69.2%). PLS-DA indicate myo-inositol, proline and glycine as additional statistically significant serum metabolites (Table 2).

**Table 2:** Statistically significant blood plasma, serum and urine metabolites according to the VIP scores of PLS-DA (VIP > 1)

Biofluid	*δ* ^1^Η (ppm)	VIP scores	Metabolites
Plasma	3.71	7.16	Glucose, leucine, cholesteryl ester
	3.85	6.99	Glucose, serine, GlcNAc
	3.67	5.54	GlcNAc, glycerol
	3.73	4.66	Glucose, alanine, cholesteryl ester
	1.29	4.28	–(C**H**_2_)_n_- fatty acids
	1.33	3.68	Lactic acid
	3.79	3.56	Glucose, lysine, GlcNAc
	3.83	3.46	Glucose, serine, sugar
	1.27	3.39	–(C**H**_2_)_n_- fatty acids
	1.31	3.33	–(C**H**_2_)_n_- fatty acids
Serum	2.05	4.83	Proline, GlcNAc
	3.67	4.82	Glycerol
	3.89	4.26	Unassigned
	3.85	3.78	Serine, glucose
	3.57	3.71	Glycine, glycerol, glucose
	1.29	3.68	–(C**H**_2_)_n_- fatty acids
	3.65	3.44	*Myo*-inositol
	3.73	3.38	Alanine, glucose
	3.79	3.34	Lysine, glucose
	3.87	3.33	Unassigned
Urine	7.67	*2.31*	3*-*methylhistidine
	3.37	2.30	Unassigned singlet
	8.23	2.29	Metabolite with substituted imidazole ring
	7.85	2.13	Hippuric acid, urocanic acid
	8.13	2.06	Metabolite with substituted imidazole ring
	7.31	1.93	Urocanic acid, metabolite with substituted imidazole ring
	3.89	1.92	Mannitol, unassigned
	7.59	1.91	Metabolite with substituted imidazole ring
	8.27	1.90	Metabolite with substituted imidazole ring
	8.01	1.89	Metabolite with substituted imidazole ring

**Fig. 2:**
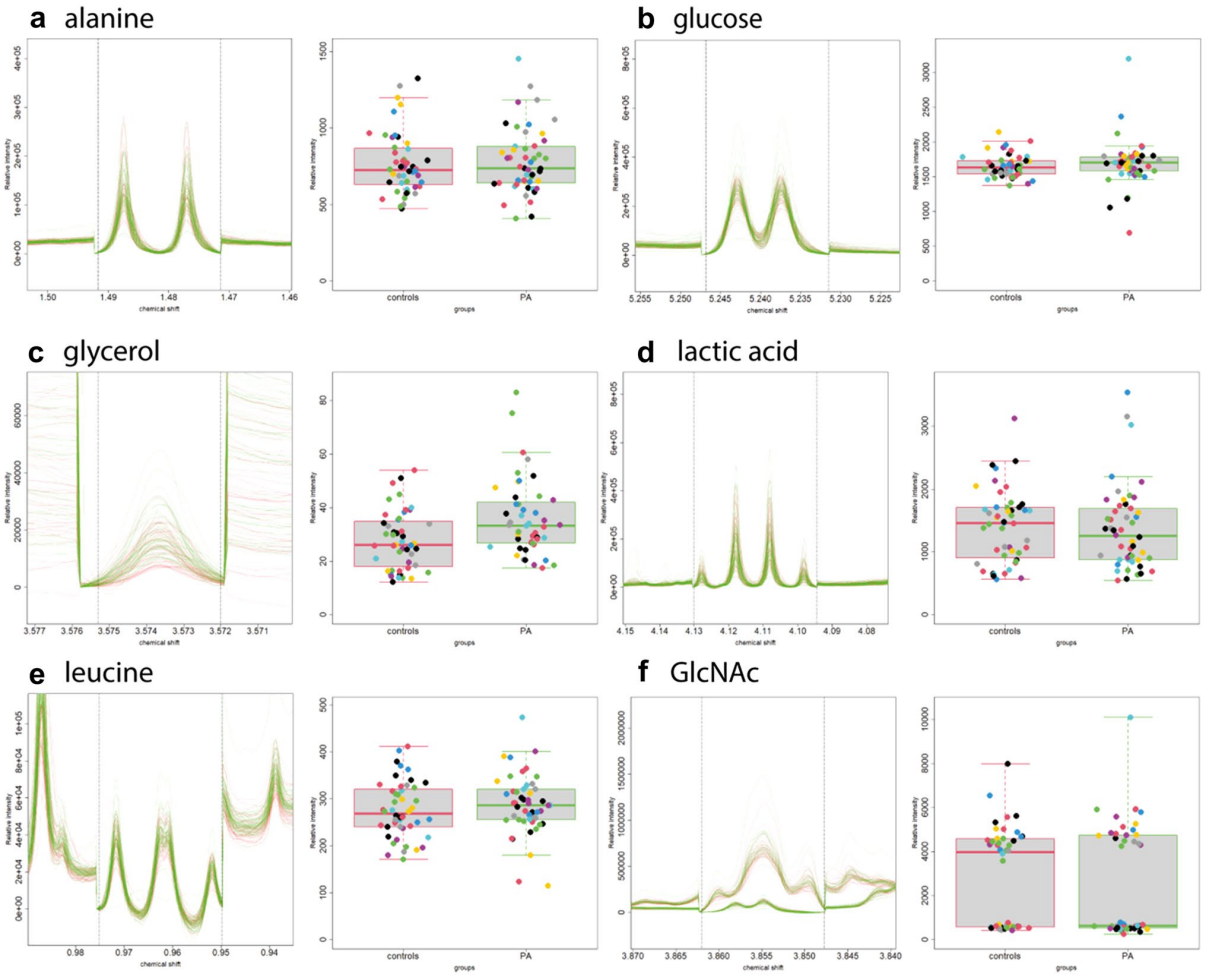
^1^H NMR signals in superimposition (left) and boxplots (right) of plasma metabolites derived by univariate analysis. Left figures: Spectral superimposition of plasma ^1^H NMR peaks for each examined metabolite (y-axis: relative intensity (a.u.); x-axis: δ ^1^Η (ppm)). Red and green ^1^H NMR spectral peaks correspond to the control and PA group, respectively. Right figures: Boxplots derived by univariate analysis for each one of the examined plasma metabolites (y-axis: relative intensity (a.u.); x-axis: group title, boxplot dots: each dot represent the.^1^H NMR spectrum of each child in different color)

The boxplots and the mean log_2_-fold changes clarified the variation of nine out of the eleven metabolites subjected into univariate analysis. Specifically, glucose, leucine, glycerol, and alanine are increased in plasma metabolic profile of children with PA, while lactic acid and GlcNAc are decreased (Fig. 2, Table 3). Furthermore, glucose, myo-inositol and serine are increased in serum spectra of PA, while glycerol and glycine are decreased in this group (Fig. S2, Table 3). Plasma and serum lysine, plasma serine and serum proline were excluded from the targeted analysis (univariate) due to high ^1^H NMR signals overlap.

**Table 3:** The mean log_2_-fold change of blood plasma and serum metabolites (PA over control) levels (Fig. S1, Fig. S2)

Biofluid	Metabolite	Mean log_2-_fold change (PA/control)	In PA children
Plasma	Alanine	0.019	**↑**
	Glucose	0.011	**↑**
	Glycerol	0.382	**↑**
	Lactic acid	− 0.071	**↓**
	Leucine	0.049	**↑**
	GlcNAc	− 0.435	**↓**
Serum	Glucose	0.021	**↑**
	Glycerol	0.084	**↑**
	Glycine	− 0.002	**↓**
	*Myo*-inositol	0.008	**↑**
	Serine	0.027	**↑**

Metabolites’ fold changes are presented using the calculated metabolite peak area in NMR spectra of PA and control group (Δp-PA/control)

↑Increased in children with PA

↓Decreased in children with PA

### Urine samples

Comparison of urine ^1^H NMR metabolic profile of PA children versus controls through PLS-DA, revealed a better clustering unlike the blood derivatives (Fig. 1a R^2^Y (cum) = 69.3% and Q^2^Y (cum) = − 42.3%). The VIP scores of PLS-DA indicate that 3-methylhistidine, hippuric acid, mannitol and urocanic acid are statistically significant for group classification. The above metabolites are decreased in children diagnosed with PA. VIP scores’ result and classification values of the PLS-DA model suggest that untargeted analysis and the whole urine metabolic fingerprint contribute significantly to class identification (Table 2).

### Correlations with androgens, BA, BMI and BW

**Fig. 3:**
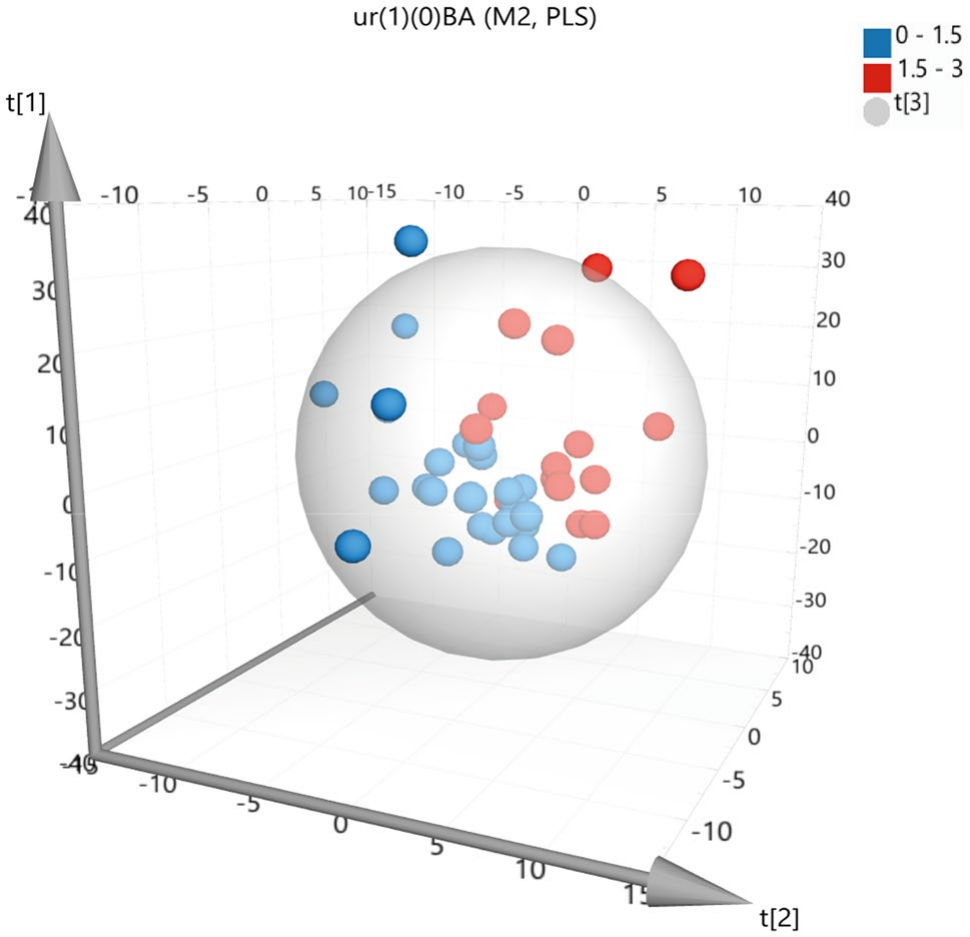
3D PLS scores plot of 40 PA urine metabolic profiles. ^1^H 1D NOESY NMR spectra distributed according to the BA (years) of the corresponding child. Urine metabolome of children with PA and BA < 1.5 years are presented in blue, while urine metabolome of children with PA and BA ≥ 1.5 years are in red

We subsequently studied, within the PA group, the correlation of the NMR metabolic profiles with androgens in serum (DHEAS, Δ4Α, 17-ΟΗP), bone age and the anthropometric data (BMI and BW). While NMR metabolic profiles in children with higher androgen levels tend to differentiate from the metabolic profiles of the control group, no distinct classification was achieved. Even though BA is not a parameter that metabolically could lead to a distinct categorization of the PA blood metabolic profile, clinically is an association factor underlying PA pathophysiology. Since BA in prepubertal children is related to obesity (Kwon et al., 2017) and childhood obesity provide a specific urine steroid profile (Gawlik et al., 2016), we attempted to test the BA as a correlation factor. When the correlation of BA with the urine ^1^H NMR metabolic profiles of PA children was examined, it was observed that urine ^1^H NMR spectral profiles of children with advanced BA by more than 1.5 years, presented a tendency for clustering in the PLS multivariate space (Fig. 3 R^2^Y (cum) = 93.6% and Q^2^Y (cum) = 3.96%). Additionally, it is acknowledged that high BMI values are positively correlated with elevated levels of blood glucose (Ortmeyer et al., 1993). Standing on this, all (control and PA children, n = 100) serum and plasma ^1^H NMR profiles were divided into four groups according to their BMI SDS values and univariate analysis was performed to investigate the glucose and myo-inositol levels variations. Glucose and myo-inositol are increased in the PA and control children with BMI SDS > 1.5 when they are compared with the PA and control children with BMI SDS ≤ 1.5, respectively (Fig. S3, Table 4). Finally, BW did not have any significant association with children’s plasma, serum, or urine ^1^H NMR metabolic profiles.

**Table 4:** The mean log_2_-fold change of blood plasma and serum glucose and myo-inositol levels (Fig. S3)

Βiofluid	Μetabolite	Mean log_2-_fold change (high/low)	BMI SDS > 1.5
Plasma PA	Glucose	0.167	**↑**
	*Myo*-inositol	0.148	**↑**
Plasma control	Glucose	− 0.029	**↑**
	*Myo*-inositol	− 0.775	**↑**
Serum PA	Glucose	0.012	**↑**
	*Myo*-inositol	0.166	**↑**
Serum control	Glucose	0.081	**↑**
	*Myo*-inositol	0.196	**↑**

Metabolites’ fold changes are presented using the calculated metabolite peak area in NMR spectra of BMI SDS > 1.5 (low) and BMI SDS ≤ 1.5 (high) (Δp-high/low)

## Discussion

To date, this study is the first NMR-based metabolomics study of children with PA and age-matched healthy control children. Current results shed light on the relationship between this endocrine disorder and its impact on metabolic profile. The metabolic fingerprint of children with premature adrenarche is differentiated from the control group according to quantitative changes in certain metabolites. More specifically the findings highlight that in all three biofluids analyzed, plasma, serum and urine, PA’s metabolic profiles show differences mainly on glucose, myo-inositol, amino acids, a population of unsaturated lipids and esterified cholesterol. Most of the statistically significant metabolic alterations belong to the glucose-alanine pathway, and lipid metabolism.

The above changes in metabolites lie within the context of a complex interplay among insulin, glucose metabolism and inositol-related molecules. Myo-inositol in the form of phosphate derivatives (inositol phosphoglycans) exerts an insulin-mimetic activity, acting downstream of insulin receptors to reduce hyperglycemia and promote muscular gluconeogenesis (Huang et al., 1993; Ortmeyer et al., 1993). Insulin resistant states followed by increased fasting glucose levels, reduce the levels of myo-inositol in serum and affects the intra/extracellular ratio of inositol molecules, contributing to metabolic dysregulation (Bletsa et al., 2021; Prieto, 2008). These metabolic interactions are tightly linked to changes in plasma and urine myo-inositol levels (Croze et al., 2015).

Previous studies have correlated the glucose, myo-inositol, fatty acids and amino acids levels changes to increased insulin levels in children and adolescents with obesity, as well as PCOS in girls (Cree-Green et al., 2019; Martos-Moreno et al., 2017). Components of metabolic syndrome such as increased fasting glucose levels accompanied by hyperinsulinemia have been reported by two independent research groups in children with PA (Ibáñez et al., 1997; Utriainen et al., 2007). Data presented herein, clearly show that both, obesity, and PA, have an impact on metabolites like glucose and myo-inositol. Investigation of BMI as a response-driven factor on metabolic profiles, showed that data analysis’ results are in line with the association between obesity and insulin resistance, suggesting a positive correlation of BMI with the glucose levels (Corvalan et al., 2013; D'Adamo et al., 2009). Interestingly, our study shows that the effect of premature adrenarche on glucose and myo-inositol levels is much more prominent in children with PA than controls, with the increase of BMI. Therefore, it seems that premature adrenarche may enhance the BMI–driven increase of metabolites like glucose and myo-inositol and deteriorate the metabolic dysregulation. In clinical practice, this emphasizes the importance of weight loss in children with PA to ameliorate its metabolic consequences.

The study of untargeted NMR-based metabolomics in urine suggests that this biofluid may be considered as the best to characterize in depth and monitor the PA, as its metabolic fingerprint is clearly reflected on urine. The metabolites 3-methylhistidine, hippuric acid, mannitol and urocanic acid are statistically significant decreased according to the supervised method PLS-DA for this clustering of urine samples. Similar urine metabolic profiles have been reported by others showing that the decreased urinary excretion of hippuric acid distinguishes adolescents with obesity from normal weight ones (Cho et al., 2017). Both urocanic acid and 3-methylhistidine are part of histidine metabolism which is strongly associated with body inflammation (Gibbs et al., 2008; Niu et al., 2012). Studies conducted in both humans and animals have shown a significantly negative correlation between histidine levels (plasma and serum) and inflammatory conditions (Holeček, 2020; Niu et al., 2012; Sun et al., 2014). Children with PA are characterized by high risk of obesity, insulin resistance, cardiovascular disease, and PCOS, which are all linked to inflammation (Ibáñez et al., 2002; Utriainen et al., 2010). In the current study, urine ^1^H NMR metabolome may be indicative of early subtle inflammatory state in children diagnosed with PA. Plasma and serum ^1^H NMR metabolome also supports the inflammatory profile through the dysregulation of glucose and myo-inositol levels. Children with PA may thus be at higher risk of developing metabolic syndrome later in life.

We should also note that the present study involved a limited number of boys with PA. Indeed, the female gender is the dominant group in our PA sample group. Although this may be considered as a limitation which could lead to a sex-biased interpretation of the PA metabolic signature, agrees with the 9:1 female to male ratio in children population that exhibit PA. Despite urine’s complexity as a biofluid, NMR metabolomics show greater sensitivity when applied in PA classification.

## Conclusion

In conclusion, urine ^1^H NMR metabolic profile provides a better clustering tendency between PA children and controls than plasma and serum ^1^H NMR metabolic profiles and considering its non-invasive collection, urine could be easily further evaluated to establish its diagnostic potential for this specific disorder. The dysregulation on glucose pathway and the inflammatory profile depicted in PA’s urine metabolome, as examined by high-resolution NMR spectroscopy might become a monitoring tool for the progression of metabolic syndrome and its complications, as the children grow through adolescence to adult life.

## Supplementary Information

Below is the link to the electronic supplementary material.

Supplementary file1 (PDF 691 kb)

## Data Availability

The datasets generated during and/or analysed during the current study are available from the corresponding author on reasonable request. NMR data have been uploaded in their final form to MetaboLights database (https://www.ebi.ac.uk/metabolights) with accession number MTBLS2387 (Haug et al., 2013).
