# Supplementary material for: NMR-based metabolic profiling of children with premature adrenarche
Source: Metabolomics. 2022 Oct 14;18(10):78. doi: 10.1007/s11306-022-01941-4 (PMC9568450; doi:10.1007/s11306-022-01941-4)
Supplement: Supplementary file 1 — Supplementary file1 (PDF 691 kb) [file 11306_2022_1941_MOESM1_ESM.pdf]

NMR-BASED METABOLIC PROFILING OF CHILDREN WITH PREMATURE ADRENARCHE

Konstantina Matzarapi[1], Aristeidis Giannakopoulos[2], Styliani A. Chasapi[1], Dimitra Kritikou[2], Alexandra Efthymiadou[2], Dionisios Chrysis[2], Georgios A. Spyroulias[1]*

[1]Department of Pharmacy, School of Health Sciences, University of Patras, Rio, 26504, Greece

[2]Division of Endocrinology Department of Pediatrics, Medical School, University of Patras, Rio, 26504, Greece

*Corresponding authors: Georgios A. Spyroulias (G.A.Spyroulias@upatras.gr)

Supplementary Material

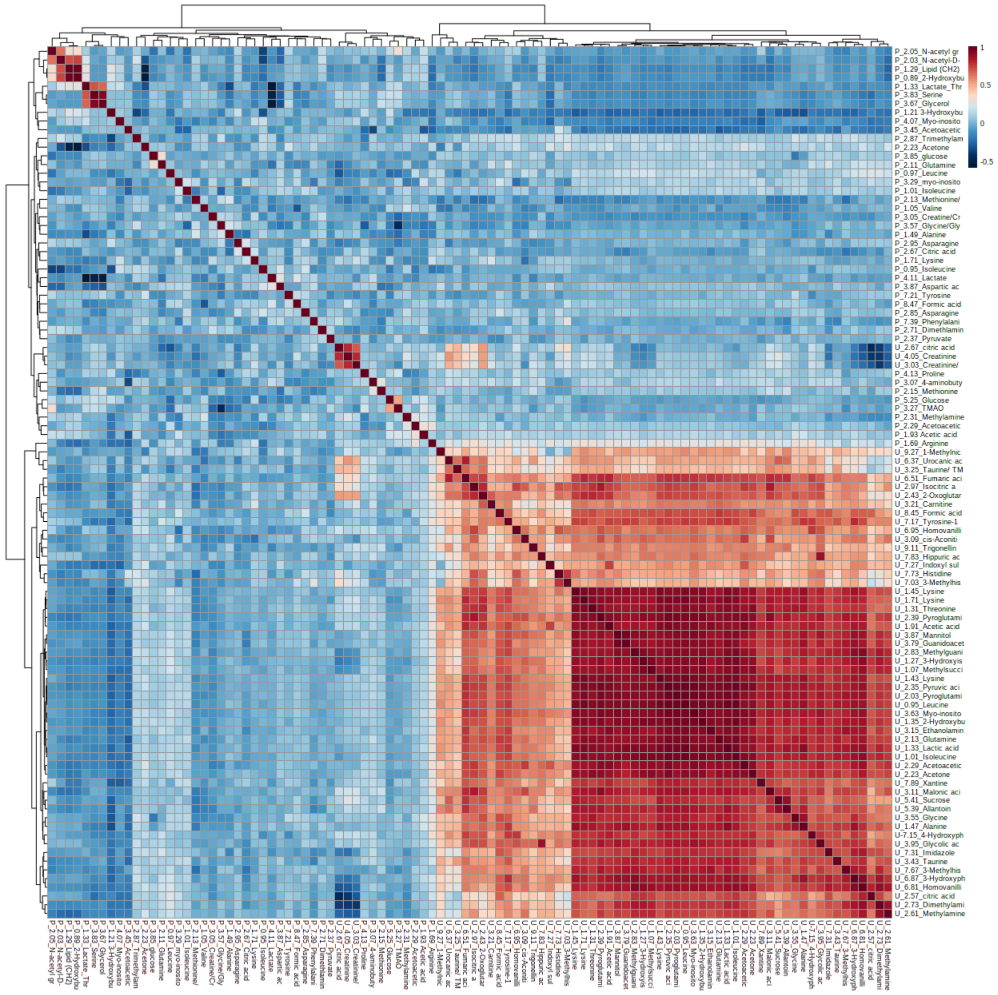

Fig S1 Hierarchical Cluster Analysis (HCA) and heatmap metabolite-metabolite correlation matrix between the indicative plasma and urine metabolite levels. The left and upper axes correspond to the HCA by which all rows and columns are sorted. Each square indicates the Pearson's correlation coefficient of the pairs: plasma -plasma, plasma-urine and urine-urine metabolite. Two main and distinct clusters emerge for urine (bottom right) and plasma (top left) metabolites, indicating the low relevance amongst them.

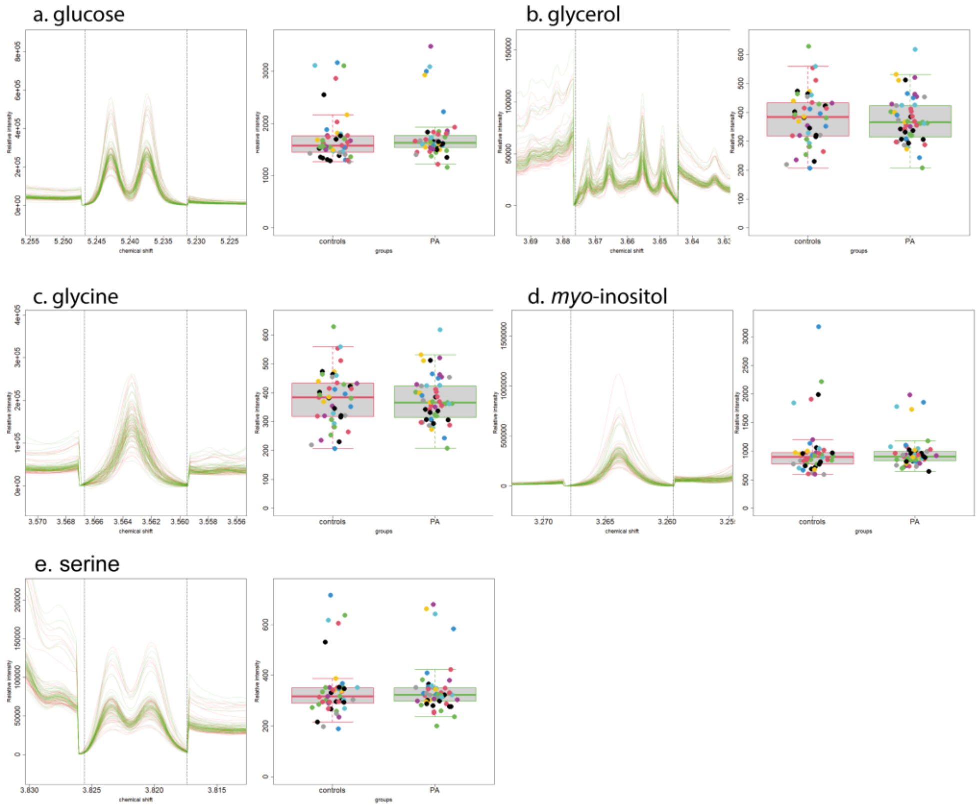

Fig S2 ^{1}H NMR signals in superimposition (left) and boxplots (right) of serum metabolites derived by univariate analysis. Left figures: Spectral superimposition of serum ^{1}H NMR peaks for each examined metabolite (y-axis: relative intensity (a.u.); x-axis: δ ^{1}H (ppm)). Red and green ^{1}H NMR spectral peaks correspond to the control and PA group, respectively. Right figures: Boxplots derived by univariate analysis for each one of the examined serum metabolites (y-axis: relative intensity (a.u.); x-axis: group title, boxplot dots: each dot represent the ^{1}H NMR spectrum of each child in different color)

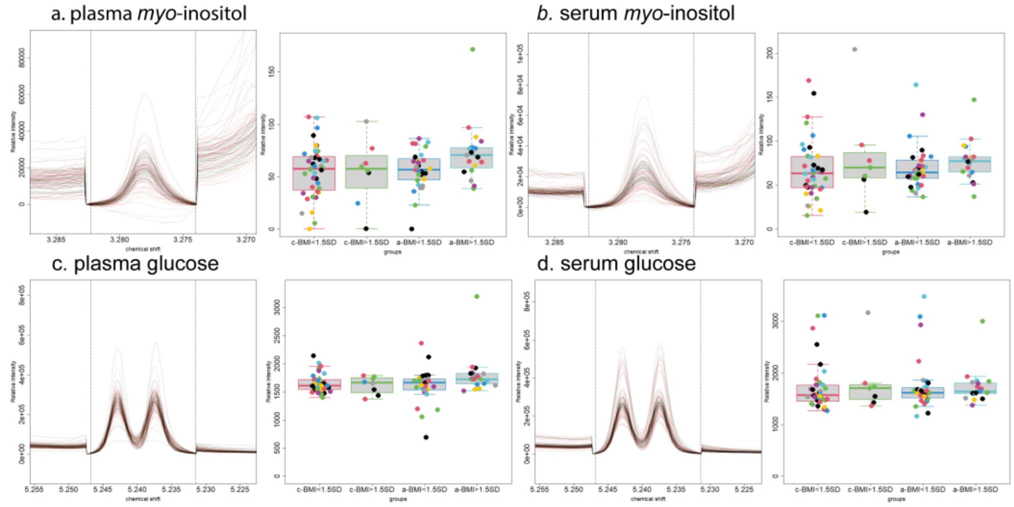

Fig S3 Boxplots and ^{1}H NMR peaks of plasma and serum glucose and myo-inositol of PA and control children used for integration. The categorization is based on their BMI SDS values. a. plasma myo-inositol; b. serum myo-inositol; c. plasma glucose; d. serum glucose; The annotation of the groups in the boxplots is explained follows: c-BMI<1.5SD: controls with BMI SDS ≤ 1.5, c-BMI>1.5SD: controls with BMI SDS > 1.5, a-BMI<1.5SD: PA with BMI SDS ≤ 1.5, a-BMI>1.5SD: PA with BMI SDS > 1.5
